# 12 Tips for taking a Student Led Medical Education Program from Concept to Curriculum

**DOI:** 10.15694/mep.2019.000190.1

**Published:** 2019-10-10

**Authors:** Thomas Sebastian Haupt, Todd Dow, Michael Smyth, Alysha Roberts, Kavita Raju, J. Thomas Toguri, Anna MacLeod

**Affiliations:** 1 Dalhousie Medical School; 2 Dalhousie Medical School

**Keywords:** Medical education, pre-clerkship, twelve-tips, physician shortage, curriculum, career decisions, student-led

## Abstract

This article was migrated. The article was marked as recommended.

The choice of a future career specialty has always been a stressful decision for medical students. To mitigate this stress and assist students in making more informed career decisions we developed the Pre-clerkship Residency Exploration Program (PREP), a two-week summer elective program that provides students with the opportunity to gain exposure to specialities that traditionally do not receive a lot of attention in medical school. To initiate this student led program we faced many obstacles, suffered many failures, learned a tremendous amount and eventually found success. In this article, we offer 12 tips on how to create a medical education program that is sustainable, effective and receives strong buy-in from faculty and administration. Our tips come from the perspective of students starting their own program but are translatable to anyone interested in taking an innovative idea and seeing it through to fruition.

## Introduction

Students have a looming fear of being denied entry into a residency program. This is because many well-known specialities are very competitive. At the same time physician shortages exist in fields such as radiation oncology and pathology ( Robboy *et al*., 2013; Yang *et al*., 2014). A challenge with medical school today is that many specialities do not receive adequate exposure in the classroom and the clinic. This lack of exposure creates uncertainty, misconceptions, stigma and ultimately results in physician shortages ( Ford, 2010). We are in a situation where students are being forced to commit earlier to a career without knowing the breadth of the specialties available to them. Society needs more physicians in these underserved specialties which are not attracting enough medical students to fill their ranks. For example, the rate of cancer is increasing and the specialists who treat it are not filling their residency spots ( Stuckless *et al*., 2012). The alarm bells have already sounded, are we listening?

With this in mind, we started the Pre-clerkship Residency Exploration Program (PREP). We constructed this program based upon feedback from students in clerkship and final year, residents, physicians, professors, and the literature. We used the Kirkpatrick model, to evaluate our program ( Praslova, 2010). PREP is a student-led two week (80 hr) intensive elective that exposes 40 medical students to 14 different specialties that students might have otherwise never experienced during the formal curriculum. To our knowledge, PREP is the largest structured elective program in Canada, with over 150 staff, faculty, and administration directly involved. Such an intricate program, with so many moving parts, took an exceptional amount of buy-in, determination and perseverance to initiate. However, within one year we were able to take our idea and establish a successful, sustainable program that effectively meets its goals. We provide herein 12 tips on how to take your medical education idea and turn it into an established, effective program.

## TIP 1: Ensure your idea is desired and valuable

In this age of information, a literature search is an important first step. A literature search may identify an existing program or framework that you can adapt and use as a model to meet your objectives, rather than starting from scratch. To do a proper literature search, one ought to answer 3 basic questions ( Swanberg *et al*., 2016):

1. What am I looking for, the more specific the better?
2. Where can I find this information?
3. Who can help me refine my search (e.g. librarian)?

At the outset, we examined the medical curriculum and elective programs offered in all 17 Canadian medical schools. We accomplished this by doing an informal search of their respective webpages. Next, a formal search with the assistance of a university librarian was performed to explore the existing literature on our idea. Librarians are experts at conducting literature searches and can aid in planning, developing, refining and reporting ( Swanberg *et al*., 2016).

Once you have relevant information on the problem you are trying to solve, you should gauge the desire from your potential participants. To assess the desire of our program we asked our class if they would find value in a program like PREP, if it were available to them. In our circumstance, there was an overwhelming response, with approximately 70% claiming they would sign up. Using this information, we built a case to garner support from student affairs, physicians and the Undergraduate Medical Education Department.

## TIP 2: Assemble a team that works well together and differ in their strengths

The culture of medicine has shifted to an interdisciplinary team-based approach. The collaborative approach applies to almost every domain in medicine and is a cornerstone of medical education and good patient care ( Burm *et al*., 2019). Learning in interprofessional teams is an important skill that fosters medical practitioners to work better together ( Hammick, Olckers and Campion-Smith, 2009).

Our team was initially three students with diverse skillsets and a common purpose. A common mistake of team building is having a collection of individuals who think alike. The best team members have strengths that compliment your weaknesses *.* Selecting roles and responsibilities based on skillsets allows for individuals to maximize potential ( Banerjee *et al*., 2016). Assemble people who have a proven ability to work in a team setting. A well-functioning team increases productivity and efficiency ( NeSmith *et al*., 2013). Regarding PREP, our team was composed of reliable individuals with the proven ability to effectively delegate work and accomplish tasks they were assigned.

The value of communicating well should not be overlooked *.* The success of any project depends on clearly communicating objectives and expectations. Most of the challenges we faced were due to a breakdown in communication. Alternatively, when we remained connected and understood the expectations of the group, each member succeeded in completing their tasks. Lastly, team members ought to exhibit flexibility.Starting a new program will not be your only responsibility. As medical students, residents, administrators or attending physicians you will be required to balance the demands of your work, volunteer commitments, family obligations and more.

## TIP 3: Establish your short-term and long-term goals early but do not hesitate to revise

As soon as a goal is developed the most important question to ask is ‘How will we meet this goal?’ A goal without a plan is a delusion and will ultimately fuel disappointment. When designing PREP, we used the SMART criteria to help us complete our objectives ( Bowman, Mogensen, Marsland and Lannin, 2015).

For PREP, we had the specific goal of obtaining participation in the program from each clinical department (e.g. Pathology, Hematology, Radiation Oncology etc.). We set a minimum target of medical disciplines as the measure of success.

With enough departments on board, we then assessed for feasibility. How many departments are required to create value for the students? How many students can be assigned to the departments? Having SMART objectives helped us focus our goals in a clear, organized fashion.

## TIP 4: Over-prepare for your consultation with key stakeholders

Given the amount of planning and preparation involved in initiating a project, an important aspect is establishing stakeholder buy-in ( Timmings *et al*., 2016). Regardless of where you reside in the hierarchy of medicine, when readiness for change exists, stakeholders are more likely to accept the change ( Jennett, Gagnon and Brandstadt, 2005). Grass roots movements with pressure for change can be more effective than top down approaches ( Jippes *et al*., 2013).

Ensure there are people at all levels ready to invest in the change, if faculty are not invested and do not see the value in the change, it may hamper your efforts for success ( Weiner, Amick and Lee, 2008). It is wise to make your first pitch to a faculty member or group that will likely aid or support your endeavour. This person might provide constructive criticism and insight to assist you in the future.

When you meet with the stakeholders, ensure you have a thoughtful, concise and well-organized presentation with ample evidence to support a defensible decision on why this program will improve medical education. Ideally, choose a team lead with a strong understanding of the subject matter and good public-speaking skills. The focus of your pitch should answer the questions “why is this program important?” and “why should I commit my time to supporting it?”

## TIP 5: Create a “win-win” situation for you and the institution

As our program targeted specialties that are not a mandatory part of the core clerkship curriculum, it was especially important to seek out leaders in departments that were looking for more student exposure. One of the goals of medical educators’ is to help students make decisions that are appropriate for them ( Reed, Jernstedt and Reber, 2001). Finding leaders that promote and share your vision is essential for any program.

In PREP for example, it was important to find leadership from each medical department that wanted to have more student exposure. We had success once we found those vital leaders in each department who could run the workshops, electives and lunchtime discussions. This was not easy, and we received as many rejections as successes.

Ultimately, this was a “win-win”, because Departments can feel more at ease that their specialty is gaining exposure and students get the opportunity to see unexplored specialities.

## TIP 6: Identify administrative obstacles and address them in a timely fashion

Following institutional approval of the program and buy-in from stakeholders, you will be required to manage the implementation process. Administrative obstacles most likely will lead to delays or failure of your program if they are not properly considered or addressed too late. Make sure you allow for enough time to handle setbacks. Administrative obstacles exist in all domains of medicine including medical education.

Understanding the administrative processes of your institution is paramount. In the case of PREP, some examples of these obstacles included: obtaining hospital card access for participants, enrollment paperwork, ensuring students had practitioner insurance to interact with patients, booking rooms, confirming instructors, etc. Furthermore, medical programs that have satellite campuses away from the main campus can present further barriers for inclusion. We had to ensure students at both campuses could obtain comparable access to the program, which required additional transport and funding considerations.

With careful planning, proactive meetings and communication with the appropriate faculty members, many of these potential administrative hurdles can be dealt with in a timely fashion.

## TIP 7: Establish a fast and simple means of communication with program participants, medical departments and organizers

The importance of communication between different medical professionals has been documented extensively ( Shamji and Deslauriers, 2018). When organizing a medical education program, there will be many potential pitfalls. It is the leadership teams’ responsibility to communicate effectively to address these issues as they arise. When selecting the appropriate modes of communication, consider what works best for everyone (email, text message, social media, face-to-face meeting etc.).

Another aspect of good communication is establishing regular meetings that focus on progress updates and addressing new obstacles. Digital lines of communication offer the added benefit of retrospective review in future years. As new executives take over the program these lines of communication can be reviewed to see what was done in years past. In our experience meeting regularly also promoted a culture of unity and camaraderie.

Before the program began, PREP directors agreed that the quickest way to disseminate information amongst participants was the utilization of a group Facebook page. A special group was made between participants and organizers which allowed for instant messaging and major updates. All participants could see scheduling changes instantaneously. It is key to select a method of communication that suits your programs needs.

Lastly, clear communication and careful scheduling with faculty well in advance can help avoid most issues. For those unforeseen problems, or unpreventable issues, we suggest having a business continuity plan. In the event x occurs, then z should be enacted.

## TIP 8: Engage in evaluation and research

PREP was designed to aid medical student career choice through exposure to a variety of underrepresented specialities. The secondary objective was to increase interest and applications to specialities that need physicians. To see if the program met these goals, careful evaluation and participant feedback is required. For each of our research questions, we developed an evaluation survey using the following guidelines:

• Apply for ethics approval if necessary
• Conduct a literature search to see how similar programs were assessed
• Delegate research responsibilities
• Prepare your survey
• Seek out professional researchers to help scrutinize your survey before completion

Evaluating impact is a crucial step to establishing your program in the evidence-based community of medicine.

## TIP 9: Incorporate quality assurance into your surveys as a means of improvement

Defining what quality means is often subjective. In the case of medical education, quality assurance (QA) is a means of measurement and actions intended to enhance your program or institution ( Manghani, 2011). Regarding PREP, we aimed to enhance both our program and our institution’s undergraduate experience. We created a structured method to gather feedback from participants, supervisors and from the leadership itself. We accomplished this in 3 ways. We created a survey (Tip 8) asking students about all experiences the program offered. Executive members participated in the program and analyzed it from a participant perspective. We interviewed departmental heads and supervisors noting their concerns and feedback.

The feedback we gathered from all parties was analyzed and formulated into a report that was disseminated to all relevant parties. Through these means the program can be changed, restructured, or adjusted to address any issues and emphasis the highly praised aspects.

## TIP 10: Research Utilization

Research utilization is the process by which science is implemented into clinical practice ( Graham *et al*., 2006). The objective behind research is to have it utilized by society for the benefit of all. It may take years to reach the stage of research utilization, do not be discouraged.

There is no set-in stone method on how to disseminate the data coming out of your program, but we would suggest starting locally. This can start as an article in the university newspaper. Furthermore, scientific conferences provide exposure to a targeted group of likeminded individuals. At conferences, not only will you be able to present your work, but you will be able to meet people with similar interests from other institutions around the country and world. PREP is now being implemented at other institutions thanks to engaging contacts at national and international conferences. Lastly, disseminating your findings through literary journals is an option that offers access to the far-reaching worlds of the scientific community.

## TIP 11: Your time is finite so establish a succession plan for your program’s future

Whether or not you plan to run your program for an extended period, this tip applies to you. The transition from creating and running a program successfully to handing over the reigns can be challenging. Accepting the loss of control influences how easily you can make the transition out. Change is a difficult road with many obstacles along the way.

Set a foundation by creating a succession plan. This will ensure the continued success of the program once you move on to the next phase of your career. Recruit new leaders as early as possible to allow time for onboarding and teambuilding. Many successful companies argue that recognizing talent is a major component of leadership (Michaels, Handfield-Jones and Axelrod, 2001). Ensure your successors are well acquainted with all key stakeholders and contacts. Introducing junior leadership early will put new faces to the head of the program and create vital connections for its success in the future.

Developing a proper manual allows for an established framework for the new leadership to reference. Ensure the manual includes contact information, an established timeline and advice for common pitfalls. Give your new leadership autonomy, micromanaging others shows self-doubt and uncertainties regarding the competence of the incoming directors. The negativity associated with micromanagement is likely due to the lack of liberty in the new roles ( Collins and Collins, 2002). This has the potential to extinguish the passion and work ethic of your incoming leadership.

New members may be nervous to take on the new role and it can be tempting to step in and do the work for them. The final and hardest task is stepping away and allowing the new leadership to find their own path.

## TIP 12: Institutional change can come from the bottom of the pecking order

In the process of starting an educational program, you will undoubtedly meet resistance, in some cases this resistance will be present at every stage of your program’s development. The phenomenon of why organizations are resistant to change has been studied extensively ( Pardo del Val and Martínez Fuentes, 2003). Rumelt separated causes of institutional resistance into 5 categories ( Rumelt, 1995). *Distorted perceptions* mean one rejects information that is contrary to what one believes to be true ( Janis, 1972). *Dulled motivation* results from an institution’s lack of incentive to make change because it creates more work of which the benefits may not be realized for years. *Failed creative response* is when one’s institution is paralysed in making decisions because the program is introduced quickly (‘paralysis by analysis’). *Political deadlocks* such as departmental politics and people who have vested interests can create barriers, especially if your program trespasses on another department’s turf. *Action disconnect* can result from disrupting the status quo and thus routine. Changing what is known for unknown can be difficult and impede progress.

Changes can come from small unlikely places. My colleagues and I had one year of medical school training when we began the year long process of creating PREP. By following many (not all) of these tips, PREP went from 3 students to over 150 staff, involving 14 medical divisions across 7 medical departments. Despite the numerous mistakes made on this journey, PREP is now in the process of being implemented in multiple medicals school across the country.

## Conclusion

PREP is a program run by students for students. It offers an opportunity to broaden career horizons and address a looming problem of physician shortages. The 12 tips provided here offer a guide on how to take an educational program idea from inception and bring it to fruition. As we navigate our own undergraduate medical education, we hope these tips will prove useful to those with the goal of starting an education program from the ground up.

## Take Home Messages

- An educational program should solve a problem that hasn’t been addressed and is important for student development.

- Assemble a well functioning team that differs in their strengths.

- No one wants extra work added to their already busy day, so the more you and your team handle the less likely you are to have resistance from important stakeholders.

- Scrutinize your program with quality assurance measures in order to improve the program year after year.

- Medical education programs can be implemented into curriculum from unlikely sources (the student body) with the right plan and team.

## Notes On Contributors

Thomas Sebastian Haupt is the author of this paper, a third-year medical student at Dalhousie University and the co-founder of PREP.

Todd Dow is a third-year medical student at Dalhousie University and founder of PREP.

Michael Smyth is a third-year medical student at Dalhousie University and the co-founder of PREP. ORCID iD: https://orcid.org/0000-0002-3310-3802

Alysha Roberts is second-year medical student and current senior director of PREP.

Kavita Raju is second-year medical student and current senior director of PREP.

J. Thomas Toguri is a second-year medical student and current senior director of PREP.

Dr. Anna MacLeod is an Associate Professor and Director of Education Research at Dalhousie Medical School.
