# Supplementary material for: 12 Tips for taking a Student Led Medical Education Program from Concept to Curriculum
Source: MedEdPublish (2016). 2019 Oct 10;8:190. [Version 1] doi: 10.15694/mep.2019.000190.1 (PMC10712485; doi:10.15694/mep.2019.000190.1)
Supplement: Peer review history

# Reviewer report: Reviewer response for version 1

**DOI:** 10.21956/mep.19895.r30110

**Published:** 2019-11-24

**Authors:** P Ravi Shankar

**Affiliations:** 1 American International Medical University

This review has been migrated. The reviewer awarded 4 stars out of 5 I enjoyed reading this article written by medical students. The PREP program seems well conceived and key features have been described well. The choice of a residency is a major decision for doctors and one they often make with incomplete information. Some specialties are better known than others and some are glamorous due to a variety of reasons. Work hours, work-life balance, emergency calls, remuneration are some important factors. The 12 steps mentioned by the authors are practical and well described. We were taught some of these steps while initiating our curriculum innovation projects (CIPs) as a requirement for FAIMER fellowships. Assembling a team that differs in its strengths is very good advice. Often we tend to assemble a team whose members think like us and have similar opinions, likes and dislikes. I am happy to know that the program is working well and is expanding. I am not sure if the authors have described the PREP program in detail elsewhere. I am interested in knowing how the two week summer program is organized and how it ensures students are exposed to different specialties. I would like to congratulate the authors on their initiative.

Reviewer Expertise:

NA

No decision status is available

# Reviewer report: Reviewer response for version 1

**DOI:** 10.21956/mep.19895.r30112

**Published:** 2019-11-15

**Authors:** Sateesh Babu Arja

**Affiliations:** 1 Avalon University School of Medicine

This review has been migrated. The reviewer awarded 4 stars out of 5 I thoroughly enjoyed reading this paper. It is a great initiative to start the Pre-clerkship Residency Exploration Program (PREP), a two-week summer elective program that provides students with the opportunity to gain exposure to specialties that traditionally do not receive a lot of attention in medical school. Any change process follows the same process of implementation, identifying the change required, valuing or defining the vision of change required, involving all stakeholders, owning of the change by all stakeholders, gathering all resources required, celebrating small victories, implementation, and evaluation. The authors detailed the process very well. Especially the steps 8 and 9 are very important after implementation. It is a good one for academic administrators.

Reviewer Expertise:

NA

No decision status is available

# Reviewer report: Reviewer response for version 1

**DOI:** 10.21956/mep.19895.r30111

**Published:** 2019-10-19

**Authors:** PATRICIA CURY

**Affiliations:** 1 FACERES

This review has been migrated. The reviewer awarded 4 stars out of 5 This is a very interesting paper and I will suggest the tips to my Instituition. It is very interesting to know that the authors are third year medical students. I wonder if residents also participate in this project.

Reviewer Expertise:

NA

No decision status is available

# Reviewer report: Reviewer response for version 1

**DOI:** 10.21956/mep.19895.r30109

**Published:** 2019-10-17

**Authors:** BALAJI ARUMUGAM

**Affiliations:** 1 TAGORE MEDICAL COLLEGE AND HOSPITAL

This review has been migrated. The reviewer awarded 4 stars out of 5 Thanks for the invite to review this article. This article deals with the...... 1. Pre-clerkship Residency Exploration Program (PREP), a two-week summer elective program that provides students with the opportunity to gain exposure to specialities that traditionally do not receive a lot of attention in medical school. 2. Which is based upon feedback from students in clerkship and final year, residents, physicians, professors, and the literature. 3. PREP is a student-led two week (80 hr) intensive elective that exposes 40 medical students to 14 different specialties that students might have otherwise never experienced during the formal curriculum. I appreciate the efforts of the medical educators, students and faculty involved in making two weeks intensive elective PREP to gain exposure to specialties based upon the feedback. The twelve tips for initiating a program was well explained and I personally feel that the tip 8 and 9 is very crucial for any program to be implemented and scope for improvement.

Reviewer Expertise:

NA

No decision status is available

# Reviewer report: Reviewer response for version 1

**DOI:** 10.21956/mep.19895.r30108

**Published:** 2019-10-10

**Authors:** Elisabeth Schlegel

**Affiliations:** 1 Donald and Barbara Zucker School of Medicine at Hofstra/Northwell

This review has been migrated. The reviewer awarded 4 stars out of 5 I thank the authors for their tremendous effort to experiment with curriculum development and to prevail in the face of adversity. Offering best practices especially from student perspectives enrich the field and provide new insights into program development. The authors describe the journey of their project with emphasis on implementation and future perspectives, such as discussing reaching consensus with stakeholders through mindful negotiation, establishing communication, or leveraging feedback. This approach is laudable, since many programs derail at the stage of development if either the institutional buy-in or the larger outlook are missing. Finally – the authors also mention proper hand-over for transitioning leadership, which is usually an underrepresented management item. My suggestions for improvement include discussing transferability and using Kern’s curricular six-step framework. The authors might formulate examples of program goals and objectives, as well as mention the educational methods. The readers wonders, how the students were prepared for residency: Was it a work-shop type with stations? Presentations held by alumni? A 12-tip paper does not need to be specific but should point the reader into the right directions. I can envision this program being evaluated using a mixed methods approach, presenting survey data and alumni/first-year resident exit interviews.

Reviewer Expertise:

NA

No decision status is available
